# Effectiveness of a resilience school-based intervention in adolescents at risk: a cluster-randomized controlled trial

**DOI:** 10.3389/fpsyg.2024.1478424

**Published:** 2024-10-22

**Authors:** Maria Llistosella, Pere Castellví, Mercedes García-Ortiz, Griselda López-Hita, Clara Torné, Ramona Ortiz, Erika Guallart, Estefanía Uña-Solbas, Juan Carlos Martín-Sánchez

**Affiliations:** ^1^Primary Health Care, Consorci Sanitari de Terrassa (CST), Terrassa, Spain; ^2^Department of Nursing, International University of Catalonia (UIC), Sant Cugat del Vallès, Spain; ^3^Department of Medicine, International University of Catalonia (UIC), Sant Cugat del Vallès, Spain; ^4^Primary Health Care, Servei Català de la Salut, Barcelona, Spain; ^5^Primary Health Care, Hospital Mútua de Terrassa, Barcelona, Spain; ^6^Department of Basic Sciences, International University of Catalonia (UIC), Sant Cugat del Vallès, Spain

**Keywords:** adolescents, randomized controlled trial, mental health promotion, school-based intervention, resilience

## Abstract

**Introduction:**

School offers a key context to promote resilience. The aim of this study was to assess the effectiveness of a school-based resilience intervention in 578 at-risk adolescents aged 12 to 15, emphasizing the significance of resilience improving mental health.

**Methods:**

A cluster-randomized controlled trial with parallel arms was conducted during the 2021/22 academic course. A specific training of six 55-minute sessions over 6 weeks was carried out for the students. Each session consisted of 5 minutes of mindfulness, followed by 45 minutes of the corresponding activity: introduction to resilience, self-esteem, emotional regulation strategies, social skills, problem solving, community resources, and peer support. Primary outcomes were assessed by the Child Youth Resilience Measure-32 at baseline (T1), post-intervention (T2), and then at the 24-week follow-up (T3). Both groups were compared using the Student’s t-test. The effect size was calculated using Cohen’s *d* and linear regression models were used.

**Results:**

A total of 578 adolescents were included, 323 in the control group and 255 in the intervention group. Significant differences in both crude and adjusted analyses for Child Youth Resilience Measure were observed at 24 weeks follow-up, showing higher resilience for the intervention group [IG, *M* = 128.7, *SD* = 14.2; CG, *M* = 125.3, *SD* = 18.4; *p* = 0.027; *d* = 0.2; *p* = 0.043, *d* = 0.16]. Furthermore, in Depressive symptoms, lower values were found for the intervention group in both crude and adjusted analyses [IG, M = 2.3 (SD = 2.5); CG, M = 2.8 (SD = 2.5); *p* = 0.04; *d =* −0.20; *p =* 0.037, *d* = −0.18].

**Discussion:**

This study contributes to fostering resilience and positive adolescent development. It also reinforces the potential of multicomponent interventions. More continuous follow-up assessments are needed to identify possible long-term changes in resilience.

**Clinical Trial Registration:**

Identifier: NCT05133115. https://clinicaltrials.gov/study/NCT05133115.

## 1 Introduction

Adolescence is characterized by biological, psychological, and social transformations with considerable changes in emotional and cognitive development (Blakemore et al., 2007; Azpiazu Izaguirre et al., 2021), being more vulnerable to developing mental health problems (Rew et al., 2014). These adolescent-specific vulnerability factors have been increased by the effect of long-term consequences of the COVID-19 pandemic and socio-economic inequalities that increase adolescents’ risk of poverty and social exclusion and have a psychological impact on their mental health (Ryu and Fan, 2023).

The negative effect of COVID-19 on adolescent mental health was described in several studies (Gracia et al., 2021; Hermosillo-de-la-Torre et al., 2021). A systematic review published by Meherali et al. (2021) concluded that increased emotional stress, anxiety, and depression were the most common consequences of the COVID-19 pandemic (Meherali et al., 2021). Furthermore, a preliminary study of the effects of the COVID-19 pandemic on suicide attempts showed that suicide attempts among adolescents increased by 25% during the COVID-19 year (Gracia et al., 2021). However, not all risk-exposed adolescents develop psychological problems; this is where the phenomenon of resilience may emerge.

Resilience is a phenomenon observed in adverse contexts where risk factors can negatively affect psychological development (Wright et al., 2013). Resilience is defined in various ways, and there is no singular or universally recognized definition (Aburn et al., 2016). Connor and Davidson (2003) defined resilience as a psychological trait or quality that characterises individuals with an increased ability to cope with adversity (Connor and Davidson, 2003). Resilience is also defined as a dynamic process (Masten, 2001) involving the adoption of positive adaptive behaviors in response to a risky environment (Masten and Obradovic, 2006). Defining resilience as a dynamic process implies that there is an association between individual traits, the risk context, and social and psychological outcomes (Masten and Obradovic, 2006).

Resilience is a complex and dynamic process (Masten, 2001) in which many protective factors are engaged. Several protective factors have been described in the literature. For example, in the Individual and Environmental Resilience Model (IERM) described by Llistosella et al. (2022), more than 60 protective factors were identified in the literature. The IERM classifies them into (a) individual factors and (b) environmental factors and highlights them with the most scientific evidence: coping, self-esteem, emotional regulation strategies, or community resources and peer support, among others (Llistosella et al., 2022). In addition, other factors were also described in the literature, such as problem-solving (Suranata et al., 2020) and mindfulness (Tripa et al., 2020). Many of these protective factors can develop over time and modify an individual’s ability to cope with adversity (Liebenberg, 2020).

Resilience is also considered a key in the perspective of positive development in adolescents and mental health (Aburn et al., 2016; Morrish et al., 2018); this is why several resilience training programs have been conducted in different contexts and populations (Chmitorz et al., 2018). Most of the resilience-based interventions found in the literature were focused on individual protective factors. Among them, we highlight social–emotional competence (Volanen et al., 2020); self-awareness (Kuperminc et al., 2020; Tripa et al., 2020), or coping skills (Suranata et al., 2020). Concerning the protective factors related to the environment, most of the interventions focused on social and school support (Kuperminc et al., 2020) and peer relationships (Kuperminc et al., 2020; Maalouf et al., 2020). Furthermore, the cognitive problem-solving technique is one of the most used in resilience interventions (Suranata et al., 2020; Llistosella et al., 2023a).

Certain types of resilience-based interventions are significantly beneficial, in particular, interventions using multicomponent (Llistosella et al., 2023a) and cognitive behavior therapy [(CBT); Dray et al., 2017; Pinto et al., 2021; Llistosella et al., 2023a]. Given the many protective factors involved in resilient processes, other resilience-based interventions focused on social–emotional learning (SEL), counseling, or mindfulness did not increase resilience alone (Llistosella et al., 2023a). This implies that further research is needed in this field to increase the evidence on resilience-based interventions (Goldberg et al., 2019) and their impacts. Recently, two meta-analyses of resilience interventions showed that resilience interventions were only effective in adolescents, especially young adolescents (between 10 and13) and at-risk populations (Llistosella et al., 2023a), but not in the general population (Llistosella et al., 2023a) and children (Pinto et al., 2021).

Furthermore, in the majority of studies included in a systematic review conducted by Samji et al. (2022), the COVID-19 pandemic has been associated with elevated levels of depressive and anxious symptoms among children and youth, along with a concerning deterioration in mental well-being (Samji et al., 2022). Consequently, innovative approaches to promote resilience and mental well-being should be developed, especially those focusing on higher-risk subgroups (Samji et al., 2022).

Given the evidence presented, and acknowledging that approximately 70 to 80% of the population lacks sufficient mental health support (Thornicroft, 2007), fostering resilience in adolescents may be an effective strategy for coping with challenges in stressful situations, such as the COVID-19 pandemic.

In summary, resilience plays a critical role in adolescent mental health outcomes. This study assesses the effectiveness of the Fostering Resilience in Adolescents at Risk (FRAK) intervention, an innovative intervention based on the recent IERM resilience model, described above. The FRAK intervention represents a comprehensive approach grounded in the recent IERM resilience model. This multicomponent intervention integrates social and emotional learning, mindfulness practices, and various protective factors, including emotional regulation, self-awareness, social support, and problem-solving skills. Developed in alignment with the ecological framework and empirical evidence, the FRAK intervention aims to enhance protective factors identified in the IERM model among at-risk adolescents (Llistosella et al., 2022).

This study was registered in the Clinical Trials (NCT05133115. November 2021) and the RCT protocol is available for review (Llistosella et al., 2023b). The established protocol was followed, without variation in the primary and secondary outcomes. The proposed statistical analyses were followed adding the effect size calculation which was not previously taken into account. Furthermore, the sample size was set at a lower number than the final number of participants in the study. However, this was also commented on in the study protocol. There were also no variations in the implementation of the intervention.

## 2 Study objectives and hypotheses

### 2.1 Objectives

The objectives of this study were: (1) to assess the effectiveness of an intervention on resilience capacities; (2) to increase the emotional regulation strategies; and (3) to assess the association between resilience intervention and depressive symptoms in adolescents at risk aged 12-to-15 (Llistosella et al., 2023b).

### 2.2 Research hypothesis

We hypothesized that adolescents at risk between 12 and 15 years old who participated in a resilience school-based intervention would increase their resilience capacities and emotional regulation strategies compared to the control group. Resilience would also be associated with a decrease in depression symptoms in the intervention group compared to the control group.

## 3 Methods and analysis

### 3.1 Design

The study is a cluster-randomized controlled trial with parallel arms (NCT05133115. November 2021). Eligible schools were randomly allocated to intervention or control groups.

### 3.2 Participants

Participants were adolescents (boys and girls) from sixth and seventh grades (aged 12-to-15 years) in a risk context (risk of social exclusion and socioeconomic deprivation neighborhoods)-and all those who consented to participate in the project. Those who did not want to participate in the intervention activities were excluded. For more information, see the RCT protocol (Llistosella et al., 2023b).

### 3.3 Procedure

**Figure 1:**
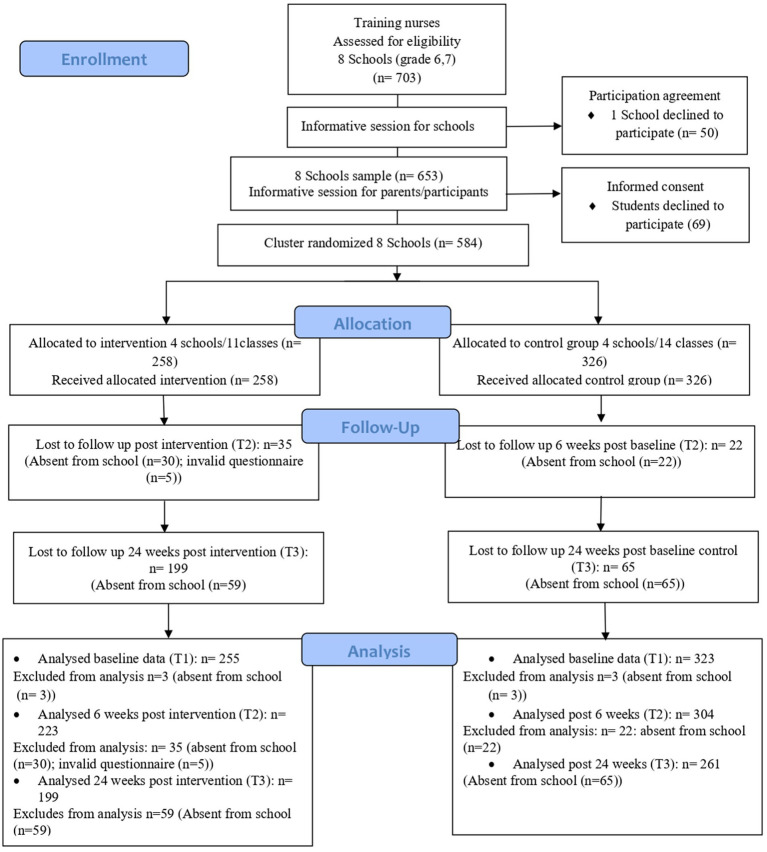
Consolidated standards of reporting Clinical Trial (CONSORT) flow chart.

Recruitment of participants began after the start of the school year (November–December 2021) in nine schools in Terrassa, Manresa, and Barcelona (in neighborhoods at risk of social exclusion), Spain. Of the nine schools contacted, one declined to participate in the project as it was already involved in another research (Figure 1).

Firstly, schools were randomly assigned to the Intervention (*n* = 11 classes) and control (*n* = 14 classes) groups by an external researcher using computer-generated random numbers; secondly, both groups were randomized into the different school sixth and seventh grades. Finally, the intervention group was composed of 7 classes of seventh grade, four classes to sixth grade, and the control group was composed of nine classes of seventh grade and 5 classes of sixth grade. This recruitment process resulted in the enrollment of 584 participants in 8 schools. Participant groups and the research team were not blinded (Llistosella et al., 2023b).

Data collection started in January 2022 and finished in December 2022. Data were collected at baseline, after 6 weeks of the intervention (Post intervention I), and 24 weeks later (Post intervention II). For more details, please see the RCT protocol (Llistosella et al., 2023b).

### 3.4 Intervention

#### 3.4.1 Intervention group

This intervention, Fostering Resilience in Adolescents at Risk (FRAK), was based on the IERM model described above and developed by a multidisciplinary and expert group. This multicomponent intervention was developed according to the empirical evidence and contextual information (Llistosella et al., 2023b). Each of the 6 weekly 55-min sessions included social and emotional learning, mindfulness, and activities to foster protective factors. Each session consisted of 5 min of mindfulness, followed by 45 min of the corresponding theme and 5 min to explain the activity to do in the reflective dossier. The sessions were outlined in the following themes: (1) introducing resilience, (2) self-esteem, (3) emotional regulation strategies, (4) social skills, (5) solving problems, (6) community resources and social and peer support. A complementary voluntary reflective dossier was designed with 6 individual activities to maintain the continuity of activities at home. For more details, please see the RCT protocol (Llistosella et al., 2023b).

Eleven primary health nurses, previously trained, carried out the intervention during school hours, and the teacher of each class was always present during all sessions. The sessions were held face-to-face and on time, despite the epidemiological situation of COVID-19. There were no significant variations during the intervention sessions.

#### 3.4.2 Control group

The research questionnaires were completed by the participants from the intervention and control groups during the same time period (January to December 2022). Following the RCT protocol and the instructions of the ethics committee, all schools in the control group were put on a waiting list to receive the intervention (Llistosella et al., 2023b). The intervention is currently being implemented in these schools.

#### 3.4.3 Monitoring procedure and risk participants

An independent monitoring committee endorsed the protocol and followed up on the process; there were no variations from the previously described protocol. During the intervention sessions, five risk cases were detected (three cases of bullying and two cases of eating disorders). Following the protocol designed (Llistosella et al., 2023b), parents and/or caregivers were informed by the nurses. A public list of resources was provided to each participant, a visit with a primary health care pediatrician was suggested, and the bullying school protocol was activated for the cases detected. Confidentiality was maintained during the process.

### 3.5 Outcomes measures

Resilience as a primary outcome was assessed using the Child Youth Resilience Measure-32 [(CYRM-32); Llistosella et al., 2019]. Additionally, the Brief Resilience Coping Scale [(BRCS); Limonero et al., 2014] was also used to analyze resilience (Table 1).

**Table 1:** Primary and secondary outcomes in FRAK study.

Outcome	Scale	Psychometric proprieties
Primary outcome		
Resilience	CYRM-32 (Llistosella et al., 2019)	A 5-point Likert-scale. 32 items designed to assess resilience in adolescents and young people (aged 12 to 23 years) in three factors (Individual Skills, Interaction with others, and Family interaction). Cronbach’s *α* was 0.88. Temporal stability was assessed by Pearson correlation and it was 0.695 for the total score of the scale. Total scores equal to or less than 132 indicate low resilience, and total scores equal to or greater than 132 indicate high resilience.
	BRCS (Limonero et al., 2014)	Self-reported measure composed of four items; for each item, participants use a 5-point Likert scale (1 = “does not describe you at all” and 5 = “it describes you well”). Cronbach’s α was 0.7; temporal stability at 6 weeks was measured by Pearson correlation, and its value was 0.69. Total scores equal to or less than 13 indicate low resilience, and total scores equal to or greater than 17 indicate high resilience.
Secondary outcome		
Emotion regulation strategies	ERQ (Cabello et al., 2013)	This questionnaire was used to assess two emotion regulation strategies: cognitive reappraisal (6 items) and expressive suppression (4 items). Participants responded using a 7-point Likert scale (1 = strongly disagree, 7 = strongly agree). Cronbach’s α coefficients were: 0.75 (Suppression) and 0.79 (Reappraisal). Pearson coefficient was used to evaluate test–retest reliability over 3 months, values were 0.66 (Suppression) and 0.64 (Reappraisal).
Depressive symptoms	Are you depressed or sad? (Chochinov et al., 1997)	Numerical scale range 0–10 (0-not depressed, 10-worst possible depression). A high sensitivity (1.00) and specificity (1.00) to identify depressed mood, and absence of false positive and negative rate (0.00) were presented.

CYRM-32, Child Youth Resilience Measure-32; BRCS, Brief Resilient Coping Scale; ERQ, Emotion Regulation Questionnaire.

Secondary outcomes were assessed with the Emotion Regulation Questionnaire [(ERQ); Gross and John, 2003; Cabello et al., 2013], and Depressive symptoms were assessed by assessing a single item: “Are you depressed or sad?” (Chochinov et al., 1997; for more information see Table 1).

In addition, demographic variables such as age, gender, and culture (European—including all countries from the European Union—and others, including Morocco, Asian countries, and Latin American countries, among others) were assessed. These variables were considered potential confounders.

### 3.6 Sample size calculation

Initially, the GRANMO tool (Antaviana, n.d.) was used to calculate the sample size with the standard deviation (SD) of the CYRM-32 scale, requiring a minimum of 70 participants per group (Llistosella et al., 2023b). However, because no randomization by classes was performed, but rather initially by schools and then by grades, as explained in the procedure section, it was decided, at the request of the schools and the ethical committee, to include all classes from each grade, resulting in a total sample of 584 participants from eight schools located in vulnerable neighborhoods; distributed as 323 students in the control group and 255 students in the intervention group.

### 3.7 Data analysis

An intention-to-treat analysis was performed. The data was described with frequency and percentage for qualitative variables, mean and standard deviation for normal quantitative variables, and median and quartiles for non-normal quantitative variables. In order to compare the two groups (control and intervention), the chi-square test was performed for qualitative variables, the t-student test was performed for normal quantitative variables, and the Mann–Whitney test was performed for non-normal quantitative variables. A regression linear model was fitted to adjust the difference between groups for gender, grade, culture, and baseline scores. To check normality, the Kolmogorov–Smirnov test and the Shapiro–Wilk test were used. All the confidence intervals will be performed with a 95% confidence level. The effect size was calculated using Cohen’s *d*. All the analyses were performed with the software R version 4.4.1 and following the research protocol (Llistosella et al., 2023b).

## 4 Results

### 4.1 Implementation

Out of 584 participants enrolled in 6th and 7th grades, 578 (98.9%) provided baseline data (T1) and were included in the analysis: 255 were assigned to the intervention group and 323 to the control group. Participants in the intervention group had high adherence: 61% (*n* = 155) attended all 6 sessions, 24.5% (*n* = 63) 5 sessions, 3.2% (*n* = 8) 4 sessions, 5.1% (*n* = 14) 3 sessions and 4.7% (*n* = 13) attended ≤2 sessions. Furthermore, 58.8% filled out the reflective dossier.

At 6 weeks of follow-up (T2), 527 (90%) participants were included in analysis. In the intervention group, 30 (11.6%) participants were absent from school and 5 (1.9%) had an invalid questionnaire; in the control group, 22 (6.7%) participants were absent from school. Finally, in 24 weeks of follow-up (T3), a total of 460 (80%) participants were included in the analysis; 59 (22.8%) participants in the intervention group and 65 (20%) in the control group were absent from school (sick, changed schools or repeated grades). Considering both groups, the missing sample did not exceed 10% in T1 and T2 and 20% in T3.

### 4.2 Baseline sample characteristics

The baseline characteristics of the total sample and in both control and intervention groups were described in Table 2. Out of the 578 participants, 46.9% were female, 72.7% were European, and 61.9% were students from 7th and 38.1% from 6th grade. The mean scores for the main variables were: CYRM-32 [M = 26.2 (SD = 16.3)]; BRCS [M = 12.8 (SD = 3.0)]; ERQ-suppression [M = 3.8 (SD = 1.3)]; and ERQ-reappraisal [M = 4.3 (SD = 1.1)]. The mean Depressive symptoms score was 2.9 (SD = 2.7). No statistically significant difference appeared in the distribution characteristics between the intervention and control groups (see Table 2).

**Table 2:** Baseline characteristics of the study population in control and intervention group.

	Overall		Control group		Intervention group		Comparison	
	*n*	%	*n*	%	*n*	%	*X^2^*	*p*
Gender							0.02	0.875
Male	307	53.1	173	53.6	134	52.5		
Female	271	46.9	150	46.4	121	47.5		
Grade							0.19	0.659
6^th^	220	38.1	126	39	94	36.9		
7^th^	358	61.9	197	61	161	63.1		
Culture	420						0.81	0.367
European	158	72.7	240	74.3	180	70.6		
Others		27.3	83	25.7	75	29.4		
	M	SD	M	SD	M	SD	*t*	*p*
CYRM-32	126.2	16.3	127.3	16.1	124.8	16.5	−1.77	0.078
BRCS	12.8	3	12.9	3.1	12.7	3	−0.71	0.476
ERQ-suppression	3.8	1.3	3.7	1.3	3.9	1.3	1.53	0.126
ERQ-reappraisal	4.3	1.1	4.3	1.1	4.4	1.2	0.85	0.397
Depressive symptoms	2.9	2.7	2.7	2.6	3	2.7	1.3	0.195

CYRM-32, Child Youth Resilience Measure-32; BRCS, Brief Resilient Coping Scale; ERQ, Emotion Regulation Questionnaire; M, mean; n, absolute frequency; *p*, *p*-value; SD, standard deviation; *t*, *t*-student test statistic; *X*^2^, chi-square test statistic.

### 4.3 Findings of the intervention

Results from 6 weeks after the intervention (T2) were presented in Table 3. No significant differences were found in all the scores for the main variables in crude and adjusted comparisons: CYRM-32 (*p* = 0.272; *p* = 0.092); BRCS (*p* = 0.697, *p* = 0.080); ERQ-suppression (*p* = 0.723; *p* = 0.193); ERQ-reappraisal (*p* = 0.723; *p* = 0.973) and Depressive symptoms (*p =* 0.954; *p* = 0.610).

**Table 3:** Outcomes at six-week follow-up in the control group and in the intervention group. Crude and adjusted comparison, including gender, grade and baseline scores of dependent variables.

	Control group		Intervention group		Crude comparison			Adjusted comparison	
	M	SD	M	SD	*t*	*p*	*d* [95% CI]	*p*	*d* [95% CI]
CYRM-32	127.0	17.9	125.3	15.9	−1.10	0.272	−0.10 [−0.27, 0.08]	0.092	−0.10 [−0.22, 0.02]
BRCS	13.1	3.0	13.2	3.0	0.39	0.697	0.03 [−0.14, 0.21]	0.808	0.02 [−0.14, 0.17]
ERQ-suppression	3.8	1.3	3.9	1.2	1.58	0.115	0.14 [−0.03, 0.32]	0.193	0.10 [−0.05, 0.25]
ERQ-reappraisal	4.2	1.1	4.2	1.1	0.35	0.723	0.03 [−0.14, 0.21]	0.973	0.00 [−0.16, 0.16]
Depressive symptoms	2.9	2.8	2.9	2.8	−0.06	0.954	0.00 [−0.18, 0.17]	0.610	−0.04 [−0.18, 0.10]

CYRM-32, Child Youth Resilience Measure-32; BRCS, Brief Resilient Coping Scale; ERQ, Emotion Regulation Questionnaire; CI, confidence interval; M, mean; *p*, *p*-value; SD, standard deviation; *t*, *t*-student test statistic; and *d* (effect size). Adjusted for gender, grade, culture, and baseline outcome.

Concerning results from 24 weeks follow-up (T3), there were significant differences in both crude and adjusted analyses for CYRM-32, showing higher scores for the intervention group [IG, *M* = 128.7, *SD* = 14.2; CG, *M* = 125.3, *SD* = 18.4; *p* = 0.027; *d* = 0.2; *p* = 0.043, *d* = 0.16]. Furthermore, in Depressive symptoms, there were lower values for the intervention group in both crude and adjusted analyses [IG, M = 2.3 (SD = 2.5); CG, M = 2.8 (SD = 2.5); *p* = 0.04; *d* = −0.20; *p =* 0.037, *d* = −0.18]. No significant difference between groups was found in BRCS (*p* = 0.229) and ERQ scales [ERQ-reappraisal (*p* = 0.256); and ERQ-suppression (*p =* 0.750); Table 4].

**Table 4:** Outcome results 24 weeks after the intervention in the control group and the intervention group. Crude comparison and adjusted comparison including gender, grade and baseline scores of the outcome.

	Control group		Intervention group		Crude comparison			Adjusted comparison	
	M	SD	M	SD	*t*	*p*	*d* [95% CI]	*p*	*d* [95% CI]
CYRM-32	125.3	18.4	128.7	14.2	2.22	0.027	0.20 [0.02, 0.39]	0.043	0.16 [0.01, 0.32]
BRCS	13.0	1.3	13.4	2.9	1.20	0.229	0.11 [−0.07, 0.30]	0.112	0.14 [−0.03, 0.32]
ERQ-suppression	3.7	1.3	3.6	1.3	−0.31	0.750	−0.03 [−0.22, 0.16]	0.669	−0.04 [−0.21, 0.14]
ERQ-reappraisal	4.2	1.1	4.4	1.2	1.14	0.256	0.11 [−0.08, 0.30]	0.333	0.09 [−0.09, 0.28]
Depressive symptoms	2.8	2.5	2.3	2.5	−2.06	0.040	−0.20 [−0.38, −0.01]	0.037	−0.18 [−0.34, −0.01]

CYRM-32, Child Youth Resilience Measure-32; BRCS, Brief Resilient Coping Scale; ERQ, Emotion Regulation Questionnaire; CI, confidence interval; M, mean; *p*, *p*-value; SD, standard deviation; *t*, *t*-student test statistic; and d (effect size). Adjusted for gender, grade, culture and baseline outcome.

Table 5 describes and compares changes in the control group and in the intervention group from the baseline (T1) to the 6-week (T2) and from the 6th week to the 24-week follow-up (T3), respectively. In the control group, the only statistically significant change was for the BRCS score in T2, although had a small increase (M = 0.4, SD = 3.0, *p* = 0.048). Regarding the intervention group, there was also a statistically significant increase in the BRCS score in T2 (M = 0.6, SD = 2.9, *p* = 0.004). In T3, the intervention group had an increase in CYRM-32 score total (M = 3.3, SD = 13.9, *p* = 0.002). Comparing the change in the two groups, there were also statistically significant differences for CYRM-32; all with a greater change in the intervention group.

**Table 5:** Change scores in the dependent variables from baseline and 6 weeks after the intervention and from 6 weeks to 24 weeks after the intervention in the control group and the intervention group and comparison.

	Control group					Intervention group					Crude comparison		
	M	SD	*t*	*p*	*d* [95% CI]	M	SD	*t*	*p*	*d* [95% CI]	*t*	*p*	*d* [95% CI]
From baseline to 6^th^ week (T1-T2)													
CYRM-32	1.0	11.7	1.35	0.177	0.08 [−0.21, 0.04]	−0.3	11.2	−0.41	0.680	−0.03 [−0.16, 0.11]	−1.21	0.223	−0.11 [−0.30, −0.07]
BRCS	0.4	3.0	1.98	0.048	0.12 [0.00, 0.24]	0.6	2.9	2.94	0.004	0.20 [0.07, 0.34]	0.84	0.404	0.08 [−0.10, 0.26]
SERQ-suppression	−0.0	1.2	−0.58	0.562	−0.04 [−0.16, 0.09]	−0.0	1.2	−0.09	0.931	0.00 [−0.14, 0.13]	0.32	0.746	0.03 [−0.15, 0.21]
ERQ-reappraisal	−0.1	1.2	−1.23	0.218	−0.08 [−0.20, 0.05]	−0.1	1.1	−1.66	0.099	−0.12 [−0.25, 0.02]	−0.33	0.742	−0,03 [−0,21, 0,15]
Depressive symptoms	−0.0	2.1	−0.03	0.977	0.00 [−0.12, 0.12]	−0.3	2.6	−1.30	0.194	−0.09 [−0.23, 0.05]	−1.05	0.296	−0.10 [−0.28, 0.08]
From 6^th^ week to 24^th^ week (T2-T3)													
CYRM-32	−0.1	15.4	−0.04	0.967	0.00 [−0.13, 0.12]	3.3	13.9	3.21	0.002	0.24 [0.09, 0.39]	2.32	0.021	0.23 [0.03, 0.42]
BRCS	−0.0	2.9	0.09	0.931	0.00 [−0.13, 0.12]	0.1	3.2	0.51	0.610	0.04 [−0.11, 0.18]	0.46	0.646	0.04 [−0.15, 0.24]
ERQ-suppression	−0.1	1.4	−0.96	0.336	−0.06 [−0.19, 0.06]	−0.2	1.3	−2.21	0.029	−0.17 [−0.32, −0.02]	−0.98	0.328	−0.10 [−0.30, 0.10]
ERQ-reappraisal	0.0	1.2	0.39	0.696	0.03 [−0.10, 0.15]	0.1	1.2	1.09	0.277	0.08 [−0.07, 0.23]	0.58	0.560	0.06 [−0.14, 0.26]
Depressive symptoms	−0.3	2.5	−1.68	0.095	−0.11 [−0.23, 0.02]	−0.4	2.8	−1.93	0.055	−0.15 [−0.30, 0.00]	−0.54	0.593	−0.05 [−0.25, 0.14]

CYRM-32, Child Youth Resilience Measure-32; BRCS, Brief Resilient Coping Scale; ERQ, Emotion Regulation Questionnaire; CI, confidence interval; M, mean; *p*, *p*-value; SD, standard deviation; *t*, *t*-student test statistic; and *d* (effect size).

## 5 Discussion

The Fostering Resilience in Adolescents at Risk (FRAK) study is the first trial developed to promote resilience in high schools for adolescents who live in socioeconomic deprivation neighborhoods in Spain.

An important outcome was that at the end of the study, higher resilience levels were obtained for the Child Youth Resilience Measure −32 (CYRM-32), along with lower levels of depressive symptoms, were observed in the intervention group compared to the control group at the 24-week follow-up (T3). Although, no significant correlation was observed with the Brief Resilient Coping Scale (BRCS) single-dimensional scale. This discrepancy may be attributed to the broader scope of dimensions assessed by the CYRM-32 scale beyond solely individual protective factors.

Contrary to our expectation, no significant difference in resilience, emotional regulation, and depressive symptoms was found immediately after the intervention (T2) between the intervention and control groups. Despite this, after 24 weeks of follow-up (T3), a significant difference was observed in resilience and depressive symptoms between groups (intervention and control), showing higher scores in resilience and lower scores in depression symptoms in the intervention group. Regarding the literature, mixed results were found in multicomponent resilience intervention studies in adolescents. On the one hand, some studies report improvements in psychological variables and resilience just after the intervention (Hyun et al., 2010; Suranata et al., 2020), and others did not (Stapleton et al., 2017). Interventions using SEL, mindfulness, or problem-solving techniques did not show changes in resilience immediately after the intervention, contrary to some studies using counselling, mentoring or CBT-based interventions (Sugiyama et al., 2020; Suranata et al., 2020; Zhang et al., 2021).

Our results did indeed report changes in resilience after 24 weeks of follow-up. Other studies with interventions similar to ours did not give short-term results (Maalouf et al., 2020; Kelley et al., 2021) but did not have a follow-up to verify whether changes occurred in the long term. In contrast, studies with interventions of 12 to 23 weeks with follow-ups for longer periods showed an increase in resilience just after the intervention (Castro-Olivo, 2014; Leventhal et al., 2015; Mirza and Arif, 2018). Multicomponent interventions based on these techniques, which involve a reflective capacity and fear on the part of the learner, likely need a certain time frame to show their effects. Consequently, more studies are required in order to determine the time of effectiveness.

Our findings showed that the improvement in resilience remained at the 24-week follow-up, suggesting that FRAK has long-term effects, although more follow-up assessments should have been done. It might have been caused by the structure of the program itself, with facilitators frequently reiterating ideas like individual strengths and valuing the strengths of others. In addition, participants were invited to apply the concepts at home and report back the following week on what they had done; the new learning was embedded and retained. Some effective interventions should include homework between sessions (Shabani et al., 2019; Helland et al., 2022).

Furthermore, the improvement in problem-solving skills was probably the result of students’ experiences in groups that often included discussions and goal-setting activities, peer relationships, transition to secondary school, and family relationships (Furness et al., 2017). Our results were also consistent with the suggestion that the development of internal assets occurs as a result of transactions between individuals and positive environmental contexts (e.g., family, school, peers, and community; Constantine et al., 1999; Kuperminc et al., 2020). It is possible that short-term improvements in external assets related to the intervention may help lay the groundwork for an eventual increase in internal assets. This suggests longer follow-ups to detect internal changes reflected in external changes and resilience. Longitudinal research with longer follow-ups is needed to test this possibility.

Regarding depressive symptoms, our results also suggested a correlation between resilience scores and depressive mood. When resilience was improved, depressive symptoms decreased, in concordance with other studies such as Sugiyama et al. (2020). Moreover, resilience is often defined as good mental health (Collishaw et al., 2016) and higher levels of resilience are associated with lower levels of depressive symptoms (Kidd and Shahar, 2008).

Contrary to our expectations, no significant differences between groups were found concerning emotional regulation strategies. We expected an increase in cognitive reappraisal strategy and a decrease in expressive suppression. This could be explained by the fact that although mindfulness was used in every session, emotional regulation was solely addressed during one session using CBT therapy (Moltrecht et al., 2021; Helland et al., 2022). Probably, more sessions and more techniques should be used to achieve a clear increase in emotional regulation strategies (Moltrecht et al., 2021; Helland et al., 2022).

Following Cohen’s criteria (Cohen, 1988), the effect size values obtained were small, but this was predictable given the characteristics of psycho-educational interventions, such as other similar studies (Díaz-González et al., 2018; Volanen et al., 2020). Probably more lasting interventions in schools in terms of time and curriculum are needed. Additionally, to continue improving resilience in adolescents, interventions specifically targeting emotional regulation strategies should be developed and assessed (Moltrecht et al., 2021), especially if they include techniques such as mindfulness-based cognitive therapy (MBCT), dialectical behavioral therapy (DBT) or acceptance-based behavioral therapy (ACT; Moltrecht et al., 2021).

### 5.1 Strength and limitations

The FRAK intervention was delivered within school hours. This represents an important strength as it does not impose personal time on the participants and their families, which could have decreased the time available for homework and increased academic stress during the intervention.

In addition, the intervention was developed by a multidisciplinary team of resilience experts and was based on an IERM resilience model and the protective factors with more evidence-based (Llistosella et al., 2022). It has also been successfully delivered to over 478 adolescents at risk, where resilience interventions were shown to be effective (Llistosella et al., 2023a).

In order to reduce bias, the nurses who delivered the intervention were previously trained using a standardized protocol, and the same nurses conducted the sessions in the same classes. Considering that one of the keys to the success of mental health promotion activities aimed at young people is the collaboration between educators and health professionals (Weist et al., 2012; O’Mara and Lind, 2013) teachers were involved in all sessions.

Although this study makes important contributions, some limitations should be considered when interpreting the results. First, the Brief Resilient Coping Scale was used to assess resilience. This scale has only four items and is a one-dimensional scale measuring individual protective factors. However, the resilience process has been described in the literature as multidimensional. Consequently, a multidimensional resilience scale was also used, namely, the Child Youth Resilience Measure-32, which includes Individual skills, Interaction with others, and Family interaction. Second, Depressive symptoms were assessed with only one item. Still, this item was used in the validation of the CYRM-32 scale in the Spanish population, giving a significantly negative correlation with resilience (Llistosella et al., 2019). Third, the sizeable attrition rate at follow-up (T3; almost 20%). However, compared with the percentage found in the literature, a retention rate of 80% is highly acceptable and similar to previous studies that have implemented this intervention (Lock and Barrett, 2003). Fourth, the RTC has been conducted exclusively within at-risk adolescents. Research indicates that resilience interventions tend to be effective only among at-risk groups (Llistosella et al., 2023a). Additionally, while all variables under investigation were measured using standardised scales, qualitative studies are needed to thoroughly examine the advantages of the FRAK intervention.

## 6 Conclusion

Adolescence represents a critical developmental phase marked by elevated stress levels, and the COVID-19 pandemic has exacerbated challenges surrounding adolescent mental health. The school-based resilience intervention in 578 at-risk adolescents aged 12 to 15 described in this article has demonstrated substantial positive outcomes, notably enhancing resilience and diminishing depressive symptoms, but only 24 weeks after it was implemented. On the other hand, it seems that it has not been effective regarding emotional regulation strategy. It is imperative to conduct extended follow-ups to ensure sustained benefits over time, as well as, to develop specific interventions to improve emotional regulation strategies in adolescents.

## Data Availability

Data will be available upon reasonable request to the corresponding author, ensuring the privacy and confidentiality of the participants.
